# Commentary: Strengths remain underweighted in dimensional and transdiagnostic models of child and youth mental health: Commentary on Altschuler et al. (2026)

**DOI:** 10.1002/jcv2.70167

**Published:** 2026-08-25

**Authors:** Toby Constable, Beth Johnson

**Affiliations:** ^1^ School of Psychological Sciences Turner Institute for Brain and Mental Health Monash University Clayton Victoria Australia

**Keywords:** psychopathology, strengths, transdiagnostic

## INTRODUCTION

This commentary considers the advances of a recent article by Altschuler et al. (2026), published in the 2026 Special Issue of *JCPP Advances*. This paper presents the case for inclusion of transdiagnostic assessment of strengths, including domains such as biopsychosocial factors, caregiver resources and needs, cultural factors and broad strengths, when evaluating child and youth mental health. Altschuler and colleague's large‐scale analysis of the Child and Adolescent Needs and Strengths Assessment, drawn from public health settings, shows how strengths among children and young people with mental health presentations may reduce the need for mental health, social, and functioning support over time. Critically, accounting for strengths and protective factors alongside standard phenotypic evaluation of mental health symptomatology within child and adolescent mental health care may help create the foundational developmental conditions needed for continuing positive outcomes for the young person.

Modern psychiatry within our healthcare systems is structured such that the need for access to psychiatric, medical or allied health services (e.g., medications, psychologists) is justified through diagnosis, and evidence of deficits and impacts on daily functioning. On the one hand, this system is designed to ensure that scarce public health resources and services are appropriately directed to support those with clear need, however this system also incentivises directing resources towards characterising deficits and risk. Altschuler and colleague's paper demonstrates that child strengths confer protection to mental health over time, and we emphasise here that omission of strengths also overlooks their key role in the aetiology and predicting prognosis of mental health conditions, as well as therapeutic recommendations during clinical formulation. In this way, Altschuler and colleague's work builds on the conversation regarding the role of strengths in child and youth mental health assessment beyond being non‐pathologising and reducing the perpetuation of stigma (Hoare et al., 2026). Their paper makes a robust case for a truly dimensional measurement of strengths across biopsychosocial functioning, family systems and support, cultural connection, and other cognitive and interpersonal strengths in understanding the aetiology of mental health presentation given the utility in outlining prognostic outcomes.

In consideration of the original article, we reflect on the value of dimensional strengths assessment across development. Altschuler and colleagues outline the complexities of diverse child presentations to mental health services. Traditional approaches within these services emphasise a deficit and needs based lens, with treatment largely involving external therapies and support provided by medical and allied health‐focused disciplines. Although strengths are strongly weighted within official biopsychosocial models of child development, in clinical practice, strengths are not consistently or comprehensively considered with respect to their role in the aetiology of child mental health, prognosis or therapeutic recommendation. As Altschuler and colleagues highlight, there is often a missed opportunity to characterise strengths and assets (e.g., family systems, talents, interests, community and coping skills) alongside vulnerabilities (e.g., mental health, social, and functioning support needs), and their paper re‐prioritises *strengths* within a biopsychosocial model of child and youth mental health. This is a timely reminder for the need to reprioritise strengths within transdiagnostic and dimensional models of childhood mental health.

Transdiagnostic and dimensional models of childhood psychopathology are well suited to capturing both strengths‐and‐deficits‐based psycho(patho)logical information, being that such nosological approaches aim to describe the full range of psychological experiences without enforcing arbitrary disorder thresholding. The Hierarchical Taxonomy of Psychopathology (HiTOP) is one such empirically derived classification system (Kotov et al., 2021). However, despite its endeavour to capture the full dimensionality of psychological traits, even in its name it is geared towards deficits rather than the full spectrum of psychological experience. Using this approach, symptoms are grouped into clusters based on patterns of item response covariation (Kotov et al., 2021). These clusters are further nested within progressively broader psychopathological dimensions, each representing an individual’s vulnerability to increasingly diffuse psychiatric difficulties. A general psychopathological health construct (the ‘*p*’ factor), reflecting an individual’s overall propensity toward psychiatric illness, sits at the apex of this hierarchy. The HiTOP approach was developed with the aim of addressing many of the well‐documented limitations of more traditional psychiatric classification systems and has been validated across a number of empirical research studies (Kotov et al., 2021).

Despite the current focus of such dimensional classifications being on psycho*pathology,* one of the greatest opportunities for these kinds of approaches to psychiatric classification is for instruments tailored to capture different ranges of the same psychological continua integrated within the same measurement model for psychiatric construct estimation (Tiego et al., 2023). Psychological strengths, including those captured by Altschuler et al. (2026), can also be organised into latent dimensional constructs via factor‐analytic (and other dimension reduction) approaches. Like psychopathological traits, psychological strengths are not unitary; they can be distinguished from one another to reflect various aspects of individual psychology contributing to wellbeing (Johnson & Wood, 2017). While such constructs are thought to fall somewhere on psychopathological continua (Keyes et al., 2005) in much the same way that the Big Five personality models have been demonstrated to do (Kotov et al., 2021), the official HiTOP model, for example, does not include such information.

Few studies have attempted to articulate the correspondence between the latent structure of psychological strengths and psychological wellbeing (e.g., that described by Gallagher et al., 2009, which also requires further independent empirical validation efforts) and HiTOP. For the majority of psychiatric measures, phenotypic resolution varies systematically with disorder severity, with most failing to adequately parse individual differences at lower ends of psychiatric illness severity. This is particularly evident in studies utilising community samples, where responses tend to be zero‐inflated (i.e., minimal‐to‐no psychopathology; Reise & Waller, 2009; Tiego et al., 2023). It is well understood that a lack of psychiatric symptomatology here would not necessarily reflect psychological resilience, for example, Incorporating instruments more sensitive to measuring these different ranges of the same psychological continua during latent factor estimation has been demonstrated to mitigate this kind of systematic signal loss (Tiego et al., 2023). For example, the Big Five, which describe typical personality variability across five validated trait domains, have repeatedly demonstrated a strong correspondence with the five‐factor model of personality disorder (Kotov et al., 2021), to the extent that they are currently thought to represent more typical ends of variation of various psychopathological continua. The official HiTOP model incorporates both Big Five and psychopathological trait data to derive its symptom clusters (Kotov et al., 2021).

Nevertheless, psychological strengths may indeed constitute the ‘flourishing’ end of various psychopathological continua as described by HiTOP. For example, the psychological strengths of pro‐sociality and community mindedness may fall on the same psychopathological spectra describing narcissistic and antisocial personality traits. If so, the direct incorporation of positive psychological constructs would accordingly yield a complete nosology; describing, as aimed, the fullest range of psychological continua from health to disorder. This would allow clinicians and researchers to describe psychological health with unprecedented precision across levels of phenotypic resolution.

We note, however, that Altschuler et al. (2026) treat psychological strengths as ostensibly separate from (albeit predictive of) psychopathological trends. Indeed, the latent structure of positive psychological traits may display correspondences with HiTOP constructs too diffuse to include as indicators for specific construct estimation. Furthermore, some research evidence suggests that psychological wellbeing and psychological ill‐health *may not* fall on the same continua (VanderWeele et al., 2026). For example, schizotypal vulnerabilities may confer a capacity for creativity (Koivisto & Toivanen, 2026), demonstrating that mental ill‐health and psychological strengths do not necessarily conform to opposite ends of the same psychological spectra. Future research is needed to disentangle the extent to which various psychological strengths reflect the inverse of psychopathological traits (e.g., as described by HiTOP) versus a complementary system for understanding and organising psychological phenomena, independent of psychiatric data, that warrants independent data collection in its own right (i.e., as demonstrated by Altschuler et al., 2026).

We further commend Altschuler et al. (2026) for their efforts to better characterise how psychological constructs unfold and differentiate over development. Many popular instruments for assessing youth mental health (including, e.g., the Paediatric Symptom Checklist and the Child Behaviour Checklist) provide age norms against which individual scores can be benchmarked. However, such instruments implicitly assume that psychopathological covariance structures remain largely invariant across child development. In other words, the constructs estimated in 18‐year‐olds are assumed to be (largely) equivalent to those estimated in 8‐year‐olds, with only minor contextualisations regarding age required for score interpretation. However, the latent structure of child and adolescent mental health may undergo substantial changes over the course of development, especially when considering strengths as well as deficits‐based information. This would be unsurprising given the scale of neurodevelopmental change occurring across this period. Accordingly, we suggest that important avenues of future research not only involve clarifying relations between psychological strengths and transdiagnostic psychopathological traits, but also how their latent structures differentiate and develop over childhood. Such research could ultimately inform the development of assessment approaches to better tease apart individual differences regardless of phenotypic resolution and identify sensitive developmental periods during which risk and resilience are most amenable to change.

## CONCLUSION

Altschuler and colleagues address a longstanding gap with respect to child and youth mental health assessment. Traditionally, diagnostic formulation and treatment planning have emphasised deficit and need, with strengths treated as secondary or foreclosed altogether from clinical interpretation. Although the inclusion of strengths during psychological assessment is not only affirming for the individual and reduces stigma, this article provides robust evidence for the role strengths play in aetiology and prognosis for youth mental health conditions. Whether or not strengths reflect psychosocial thriving along the same dimensional lines as psychopathology, form a related but distinct system of individual differences, or some combination depending on the construct, is still unresolved. Answering this will require factor‐analytic work testing correspondence with transdiagnostic models, alongside further independent validation of strengths‐based instruments. Careful consideration of developmental stage remains necessary here; it is likely that, just as the latent structure of child psychopathology differentiates over time, so too does the latent structure of psychological strengths. In addition to motivating the systematic and transdiagnostic measurement of strengths in youth mental health, this paper invites the extension of developmental and structural evidence to realise a truly dimensional model of child and youth mental health.

## AUTHOR CONTRIBUTIONS

**Toby Constable:** Conceptualization; writing—original draft; writing—review and editing. **Beth Johnson**: Conceptualization; writing—original draft; writing—review and editing.

## CONFLICT OF INTEREST STATEMENT

The authors declare no conflicts of interest.

## ETHICAL CONSIDERATIONS

Ethical approval is not applicable to this commentary article.

## Data Availability

Data availability is not applicable to this commentary article.
